# Antioxidant and Antimicrobial Effects of Polyphenolic Extracts from Olive Mill Vegetation Water on Wild Boar Meat Patties

**DOI:** 10.3390/molecules30244692

**Published:** 2025-12-08

**Authors:** Caterina Altissimi, David Ranucci, Susanne Bauer, Raffaella Branciari, Roberta Galarini, Maurizio Servili, Rossana Roila, Peter Paulsen

**Affiliations:** 1 Department of Veterinary Medicine, University of Perugia, 06124 Perugia, Italy; caterina.altissimi@unipg.it (C.A.); david.ranucci@unipg.it (D.R.); rossana.roila@unipg.it (R.R.); 2 Centre for Food Science and Veterinary Public Health, Clinical Department for Farm Animals and Food System Science, University of Veterinary Medicine Vienna, 1210 Vienna, Austria; susanne.bauer@vetmeduni.ac.at (S.B.); peter.paulsen@vetmeduni.ac.at (P.P.); 3 Istituto Zooprofilattico Sperimentale dell’Umbria e delle Marche “Togo Rosati”, 06132 Perugia, Italy; r.galarini@izsum.it; 4 Department of Agricultural, Food and Environmental Sciences, University of Perugia, 06121 Perugia, Italy; maurizio.servili@unipg.it

**Keywords:** game meat, hydroxytyrosol, tyrosol, lipid oxidation, spoilage microorganisms

## Abstract

Game meats are particularly prone to oxidation and microbial spoilage due to their specific characteristics and the procedures required to obtain them. Various sustainable bioactive molecules derived from food industry by-products, such as olive mill wastewater, have the potential to enhance the stability and safety of game meats. The use of different levels of polyphenolic extracts from olive mill vegetation water, encapsulated through a freeze-drying process, was tested on wild boar meat patties as an antioxidant and antimicrobial. Two separate trials were performed. Trial 1 was carried out by adding different concentrations of polyphenolic extract (0, 1, and 2%) during the production of wild boar patties, and trial 2 by adding 1.5% salt and adding or not adding 2% polyphenolic extract. The first trial revealed antioxidant effects on the raw patties during storage time, both on colour (increasing in saturation index) and thiobarbituric acid-reactive substances (0.306, 0.268, and 0.254 mg MDA/kg after 5 days of storage in the control with 1% and 2% polyphenolic extract groups, respectively). Oxidation was also reduced during cold storage of cooked patties. Trial 1 also revealed a dose-dependent antimicrobial effect, mainly on *Enterobacteriaceae* and *Pseudomonas* spp. Trial 2 confirmed that salt plus extract addition had an overall higher antimicrobial effect than when singularly added, but with a moderate increase in the hardness of the products.

## 1. Introduction

Extensive literature highlights the presence of polyphenols belonging to the phenylalcohol subclass, particularly tyrosol (T) and hydroxytyrosol (HT), in various plant sources, predominantly in olive-derived products such as fruits, leaves, and oil [1,2]. These compounds are known for their numerous health-promoting properties and biological activities [3]. HT and T exert potent antioxidant effects, largely attributed to their chemical structures, which enable them to inhibit the formation of free radicals generated during autoxidation processes [4]. The antimicrobial activity of olive phenols is primarily associated with their dialdehydic structure, which allows interactions with amino acids, proteins, and membrane molecules, ultimately leading to membrane permeabilization and bacterial cell lysis [5]. The olive oil industry generates considerable amounts of solid (olive pomace) and liquid (olive mill wastewater) by-products, which are rich in HT, T, and more complex polyphenolic compounds. Due to their high phytotoxicity, these wastes pose a significant environmental threat to both terrestrial and aquatic ecosystems, particularly in the Mediterranean region [6]. Consequently, there is increasing interest in their appropriate disposal and valorisation. One promising direction involves their reutilization in the food industry. Importantly, these by-products are characterized by a high content of phenolic compounds with demonstrated antioxidant and antimicrobial properties, including 3,4-dihydroxyphenylethanol (3,4-DHPEA or hydroxytyrosol) and p-hydroxyphenylethanol (p-HPEA or tyrosol). Extracts obtained from these waste materials may serve as natural alternatives to synthetic preservatives commonly used in the food industry [7].

Wild boar meat has been reported to possess superior nutritional qualities compared to conventional livestock meat [8]. However, the uncontrolled nature of its production—particularly with respect to handling, microbial contamination, and storage conditions—contributes to a higher rate of spoilage [9]. Additionally, wild boar meat is often perceived as tough and less suitable for direct consumption, leading to its frequent use in processed forms such as minced meat patties. The grinding process disrupts the muscle structure, creating a less stable matrix that promotes microbial growth as well as chemical and enzymatic oxidation. Furthermore, wild boar meat is also prone to oxidation for its high amount of polyunsaturated fatty acids (PUFAs), mostly omega 3 [8]. These changes can negatively affect the safety and nutritional quality of the final product. In the food industry, especially in fresh ground meat products, additives with antioxidant properties are routinely used to mitigate these effects [7,8].

Despite being regulated under Regulation (EC) No. 1333/2008 (and subsequent amendments), synthetic food additives are often viewed with scepticism by consumers due to concerns about their potential long-term health effects [10]. As a result, there is growing consumer demand for food products containing natural antioxidants, prompting ongoing research into natural alternatives to synthetic preservatives. Among agro-industrial by-products, liquid wastes from olive oil production—namely olive mill wastewaters (OMWWs)—have shown considerable potential [11]. OMWWs contain significant concentrations of phenolic compounds, particularly HT and T, which possess well-documented antioxidant and antimicrobial activities. These properties make OMWWs promising candidates for application in food preservation, especially in meat products susceptible to oxidative and microbial spoilage such as minced meat.

The aim of this study was to explore how the addition of different concentrations of polyphenolic extract (PPE) from olive mill vegetation water (OMWW) into wild boar meat patties would enhance microbiological and chemical shelf life, and how it would influence physical-chemical traits, such as colour, water activity, and texture properties.

## 2. Results

### 2.1. Trial 1: Effects of Two PPE Doses on Antioxidant and Microbial Contamination of Wild Boar Patties

**Figure 1:**
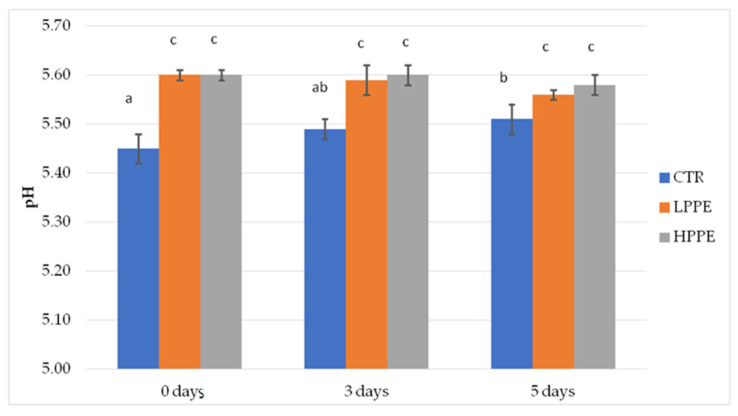
pH of wild boar meat patties with 0 (CTR), 1% polyphenol extract (LPPE), and 2% polyphenol extract (HPPE) at the day of production and after for 3 or 5 days in vac. storage at 3 °C. CTR = control group without polyphenolic extract, LPPE = group with 1% polyphenolic extract, HPPE = group with 2% polyphenolic extract. Each value represents the average of 3 measurements per sample (*n* = 81). Within columns, different superscripts (a, b, c) indicate statistically significant differences, *p* < 0.05.

The pH values recorded in trial 1 are reported in Figure 1 with no differences among LPPE and HPPE at each time (*p* < 0.05).

Water activity (a_w_) was 0.97 ± 0.001 in the control group (CTR) and the same results were found in the group with 1 and 2% PPE added (group LPPE and HPPE).

The proximate composition of the wild boar meat patties is reported in Table 1. Differences were detected only for moisture content, with lower values in the group with a higher quantity of PPE.

The evolution of instrumental colour parameters—lightness (L*), redness (a*), and yellowness (b*)—of the different patty formulations during refrigerated storage is presented in Table 2. The colour of the patties was influenced by the incorporation of PPE, particularly concerning redness and yellowness. Specifically, the a* value decreased in the CTR samples over time, while it increased in the treated samples. Similarly, the b* value remained stable in the CTR group but increased in the PPE-treated samples. At day 0 of storage, L* values were similar across all groups. However, during storage, lightness increased in both the CTR and LPPE samples, while it remained stable in the HPPE group. Additionally, the inclusion of the extract altered the initial colour of the patties, resulting in lower redness and yellowness in treated samples compared to the control. Interestingly, by the end of the storage period, the treated samples exhibited higher a* and b* values than the control, indicating a protective effect of PPE on colour stability. Likewise, chroma (saturation) increased in the treated samples during storage, whereas it remained stable in the control group. Nevertheless, the addition of phenols had no effect on hue angle.

For each group the ΔE calculated revealed perceptible changes during time, as ΔE in the CTR and in PPE groups was >2 after 3 days and >3.5 after 5 days.

The microbial counts of the control patties before vacuum packaging were 6.12 ± 0.24, 2.91 ± 0.41, and 3.20 ± 0.35 Log cfu/g for ACC, *Enterobacteriaceae*, and *Pseudomonas*, respectively. The counts determined on raw patties after 3 and 5 days under vacuum storage at 3 °C are reported in Table 3. The difference between groups was detected for all three parameters considered, with lower values of ACC in treated patties and *Enterobacteriaceae* and *Pseudomonas* spp. in patties with 2% of PPE both after 3 and 5 days of storage. An increase in counts was noticed during storage time only for ACC in all the groups and in *Enterobacteriaceae* in the control group (Table 3).

The average TBARS values after 3 and 5 days in both raw and cooked patties were higher in the control group than in treated ones. Differences due to storage time were highlighted in raw products but no differences were detected in treated cooked patties. Higher malondialdehyde (MDA) contents were observed after cooking, indicating that the heating process triggered fat oxidation (Table 4). The increase was, however, less pronounced in treated samples than in controls, confirming the antioxidant effect of PPE.

### 2.2. Trial 2: Patties with and Without PPE 2% and NaCl 1.5%

**Figure 2:**
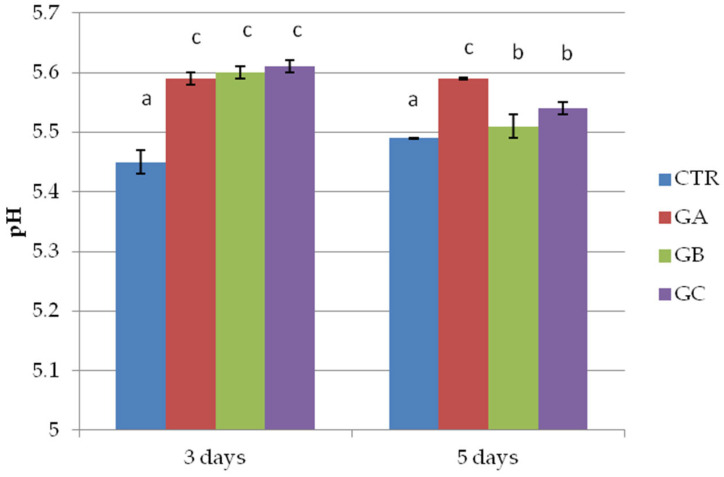
pH of wild boar meat patties with 0 and 2% polyphenol extract (PPE) and 0 and 1.5% NaCl, in vac. storage for 3 or 5 days at 3 °C. CTR = group without polyphenolic extract and NaCl, GA = group without polyphenolic extract and with 1.5% NaCl, GB = group with 2% polyphenolic extract and without NaCl, GC = group with 2% polyphenolic extract and 1.5% NaCl. Each value represents the average of 3 measurements per sample (*n* = 48). Within columns, different superscripts (a, b, c) indicate statistically significant differences, *p* < 0.05.

**Figure 3:**
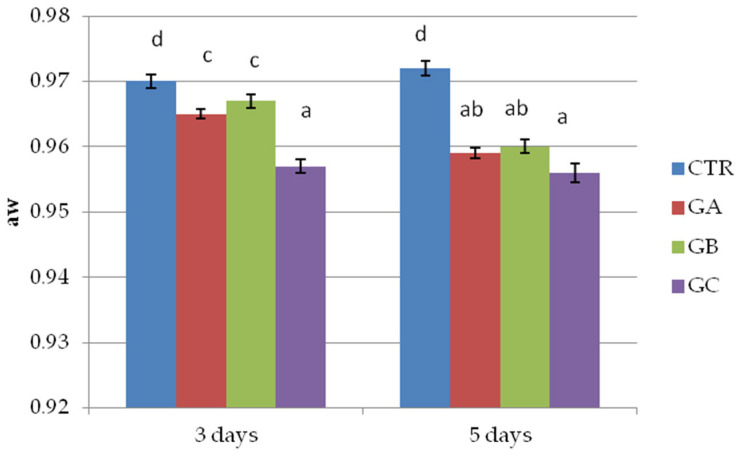
Water activity (a_w_) of wild boar meat patties with 0 and 2% polyphenolic extract (PPE) and 0 and 1.5% NaCl, in vac. storage for 3 or 5 days at 3 °C. CTR = group without polyphenolic extract and NaCl, GA = group without polyphenolic extract and with 1.5% NaCl, GB = group with 2% polyphenolic extract and without NaCl, GC = group with 2% polyphenolic extract and 1.5% NaCl. Each value represents the average of 3 measurements per sample (*n* = 48). Within columns, different superscripts (a, b, c, d) indicate statistically significant differences, *p* < 0.05.

The results of the pH measured in the wild boar patties of the second trial highlight differences between patties with no PPE and salt added and the other groups. The addition of both salt and PPE was associated with an increase in the pH values in the products (Figure 2). The a_w_ values were different between the groups, with higher values in patties made with no additives and lower values in those with salt and PPE addition (Figure 3). Differences between 3 and 5 days of storage were detected only for the patties manufactured with 2% PPE but without salt.

Development of the microflora of raw patties stored for 3 or 5 days vacuum-packaged at 3 ± 1 °C is shown in Table 5. Generally, samples with 2% PPE or 2% PPE and 1.5% NaCl added demonstrated significantly (*p* < 0.05) lower numbers of total aerobic colony count, *Pseudomonas* spp., and *Enterobacteriaceae* than controls (0% PPE, 0% NaCl), with differences in the order of magnitude of 1 log cycle in samples stored for 5 days.

Addition of 1.5% NaCl effectuated significantly higher results for hardness and toughness after 5 days of storage, whereas addition of 2% PPE had no significant effect on textural properties (Table 6). Cooking loss values were higher in samples with both salt and PPE added (*p* < 0.001).

## 3. Discussion

### 3.1. Antioxidant Activity of Phenols

The integration of phenols in wild boar meat patties ensures oxidative stability during storage time. This result can be attributed to the antioxidant capacity of PPE added into patties related to its chemical composition, as it is mainly characterized by hydroxytyrosol [7]. It has been proven that the strong antioxidant capacity of hydroxytyrosol is closely associated with its chemical structure: a phenol ring formed by a catechol group and three hydroxyl groups [12]. These functional groups could promote its preservative action in products of animal origin, as demonstrated in the literature [7,13] and confirmed in our study.

We observed a significant reduction in lipid oxidation in wild boar patties with polyphenolic extract added, especially at concentrations of both 1% and 2%, as indicated by reduced TBARS levels. This effect highlights the potent antioxidant capacity of PPE, which is crucial for extending shelf life and maintaining meat quality. The reduction in TBARSs suggests that PPE efficiently inhibited lipid peroxidation, a primary factor in the quality deterioration of meat during storage. The polyphenolic compounds in PPE, particularly hydroxytyrosol, play a significant role in the antioxidant activity. Hydroxytyrosol and other phenols are known for their ability to scavenge free radicals, interrupting the oxidative chain reaction that leads to lipid degradation [14,15]. Our findings align with those reported by Roila et al. [7], where beef burgers treated with olive mill wastewater polyphenolic extract showed up to 62% lower TBARS levels over seven days compared to untreated controls, highlighting the effectiveness of polyphenol extracts in reducing lipid oxidation. Furthermore, Martínez-Zamora et al. [12] observed a similar reduction in lipid oxidation when hydroxytyrosol was applied to lamb patties, achieving a 35% decrease in oxidation compared to control samples. Moreover, our data suggest that the antioxidant properties of PPE were effective after heating and subsequent cold storage, simulating realistic consumption scenarios for processed meat products. The addition of PPE (polyphenolic extract) caused a colour modification in the patties. The variation in colour characteristics may result from the pigments present in the extract utilized, which can substantially influence the colouration of the patties. The results of this investigation are analogous to other studies indicating a colour alteration using different extracts [16]. The study’s findings confirm that CTR patties exhibited discolouration during storage compared to treated samples, primarily characterized by a decrease in redness, and are consistent with observations by other researchers [17,18,19] regarding raw meat during refrigerated storage. The stabilizing influence of natural antioxidants on colour has been evidenced in research involving various natural antioxidants, such as avocado [19], rosemary and lemon balm [20], carnosine [21], and red grape [22]. Lorenzo et al. [23] obtained analogous results with ground pork treated with grape seed extract, where the treated samples displayed a more pronounced red colour than the control after 20 days of storage. The findings presented here align also with those of Hawashine et al. [24] and Bouraban Chibane et al. [16] about beef patties treated with olive cake, pomegranate peel, and grape seed, respectively. Furthermore, the improvement of the saturation index and the stability of the hue angle in treated samples confirmed the efficacy of polyphenols in meat for maintaining an attractive colour [25]. The discolouration noted in the preservation of red meat cuts is generally associated with the oxidation of the iron atom in the haem group of red oxymyoglobin (OxyMb) to brown metmyoglobin (MetMb) [26]. The accumulation of MetMb on the meat surface and the consequent discolouration mostly depend on the presence of reducing processes and lipid oxidation [17,26].

### 3.2. Antimicrobial Activity of Phenols

The antimicrobial activity of olive phenols is attributed to their dialdehydic structure, which interacts significantly with amino acids, proteins, and membrane molecules, leading to membrane permeabilization and bacterial cell lysis. Research indicates that tyrosol inhibits cyclooxygenase enzyme activity, while hydroxytyrosol exhibits protein-denaturing properties [27]. Additional research indicates that numerous polyphenolic compounds serve as effective iron scavengers, and insufficient iron impacts the growth of specific pathogenic bacteria by diminishing the ribonucleotide precursor necessary for DNA synthesis [28]. The inclusion of PPE showed a limited effect regarding antimicrobial activity. The use of a 2% concentration of PPE in wild boar patties led to a slight reduction in ACC and EB compared to the control, observed at both 3 and 5 days of refrigerated storage, while the reduction in PS was noted only after 3 days of storage. Similar limited antimicrobial effects of polyphenols were noted in a previous study on game meat by Altissimi et al. [29], despite variations in concentrations, application methods, and storage times. While the antimicrobial effects of PPE were modest, this aligns with previous findings indicating that polyphenols generally exert mild antibacterial activity in beef meat products [15]. The effects of the PPE application on other spoilage microorganisms, such as Lactic Acid Bacteria, have been investigated previously in similar meat preparations and were found to be negligible [7]. Notably, some studies suggest that using different concentrations of polyphenols or combining them with other natural antimicrobials, such as essential oils or organic acids, may offer a strategic approach to enhance the antimicrobial efficacy [16,30].

The combination of polyphenolic extract (PPE) with sodium chloride (NaCl) further improved the microbiological stability of the patties, effectively reducing microbial spoilage. NaCl is known to have antimicrobial effects due to osmotic pressure, and its combination with PPE appears to create an enhanced effect on bacterial inhibition. A synergistic action of salt and natural antimicrobials has been studied and appears to be a promising strategy to enhance the preservative effect [31,32]. This approach allows for a reduction in the concentration of plant-derived extracts, which at high levels could negatively affect the sensory characteristics of some products, while also decreasing the use of salt in certain meat products, thereby ensuring the safety and quality of meat products [32].

### 3.3. Effects of PPE on Wild Boar Patty Quality Traits

The quality traits of the patties were investigated mainly by colour and texture. As previously reported, the effects of the addition of PPE on the wild boar patties were significant for the colour lightness and redness; therefore, the saturation index is potentially affected by meat oxidation. Indeed, wild boar meat generally presents a different colour from pig, due to higher water retention of the muscle and amount of myoglobin [33]. From a sensory perspective, overall colour differences (ΔE) change independently within the observed group over the course of storage time, denoting that the addition of PPE cannot affect the overall colour evolution of the product. The comparison between the groups highlights that no differences in ΔE were recorded after five days and were only significant between LPPE and HPPE at three days (Table 2). The ΔE value after 3 days for LPPE is lower than the other experimental groups, attesting that this specific PPE addition allows for overall colour stabilization, making colour differences over time imperceptible to common consumers. This observation was further confirmed in frankfurter sausages treated with polyphenols, where ΔE measurements showed no noticeable colour differences for consumers, particularly in products with naturally darker hues [34].

Textural effects were observed on patties in trial 2 with an increase in the hardness and related parameters, according to the NaCl addition and the storage time of the product. These differences in the texture profiles registered (Table 6) could be ascribed to the difference in the moisture content between the control and HPPE patties (highlighted in trial 1, Table 1), due to a possible reduction in the moisture content of the products during storage time being increased by salt addition and the solubilization of the functional myofibrillar proteins due to salt that results in a tougher product [35]. Furthermore, the higher values for hardness and other textural properties in patties manufactured with NaCl are in agreement with sensory assessments (“firmness”) of coarse meat patties manufactured with varying amounts of NaCl [31]. Indeed, cooking loss was also influenced by salt addition and contributed to higher hardness values.

In conclusion, from a sensory perspective, the use of PPE in the encapsulated form on meats is particularly advantageous. Freeze-drying encapsulation with maltodextrin is demonstrated to effectively protect bioactive compounds from oxygen, light, and moisture, enhancing their stability and facilitating their incorporation into complex food systems [36]. Moreover, the use of encapsulated polyphenolic extracts has been proven not to negatively affect the sensory properties of meat products. Evidence from products such as burgers and pork patties confirm that encapsulated olive polyphenols do not induce off-flavours, bitterness, or astringency, supporting their practical feasibility and consumer acceptability [25].

## 4. Materials and Methods

### 4.1. Sample Preparation and Outline of the Experiments

We studied the effect of varying concentrations of a polyphenolic extract (PPE) from OMWWs and salt (NaCl) on pH, water activity, colour, specific microbial hygiene criteria, and thiobarbituric acid-reactive substances (TBARSs) in patties produced from wild boar meat.

#### 4.1.1. Polyphenolic Extract Characterization

The extracts, which are rich in polyphenols, were supplied by Stymon Natural Products P.C., located in Patras, Greece. This product is derived from OMWWs (*Olea europaea* L., Koroneiki cultivar). The extracts were provided as encapsulated powder obtained through a freeze-drying process. Specifically, the extracted polyphenols were subjected to freeze-drying encapsulation within amorphous carbohydrate microstructure matrices, such as maltodextrin. The product is manufactured through a patented process (GR1010150, EP4049543A1) utilizing environmentally friendly technologies. In brief, the encapsulated extract is produced by treatment with hydrolytic enzymes, followed by filtration using a membrane process, and encapsulation with a food-grade maltodextrin carrier at a 1:1 dry weight ratio. This was succeeded by lyophilization at −55 °C and 0.1 mbar, and a subsequent grinding process (freeze-drying process). The identification of single molecules composing the extract and the determination of their concentrations were carried out using Liquid Chromatography Quadrupole Time-of-Flight spectrometry (LC-QTOF; LC-TripleTOF 6600, Sciex, Framingham, MA, USA), as previously described by Roila et al. [37]. Briefly, the equipment consisted of an Exion LC™ coupled to a 6600+ TripleTOF™ (Sciex, Foster, CA, USA) equipped with an electrospray ionization source operating in negative mode (ESI-). Chromatographic separation was performed using an Acquity BEH C18 column (150 mm × 2.1 mm, 1.7 μm, Waters, Milford, MA, USA). The mobile phases were water with 0.025% acetic acid (A) and methanol/ACN 90/10 *v*/*v*% (B). Gradient elution programme: The initial mobile phase composition was 100% A held for 1 min, then linearly decreased to 80% A at 11 min, to 50% A at 15 min, and to 0% A at 18 min. This condition (100% B) was maintained until 23 min, then the system was returned to the initial conditions (100% A) within 1 min and equilibrated until 30 min. The autosampler was maintained at 25 °C, whereas the column was set at 40 °C. The injection volume was 10 µL, and the flow rate 0.25 mL/min. The curtain gas (40 psi) was nitrogen, while the GS1 (55 psi) and GS2 (55 psi) were compressed air. The interface source temperature and the spray voltage were set at 450 °C and −4.5 kV, respectively. The quantification was carried out using a five-point calibration curve (25 ng/mL, 50 ng/mL, 100 ng/mL, 250 ng/mL, and 500 ng/mL) prepared in a mixture of acetic acid 0.025%/MeOH 90/10 (*v*/*v*).

Approximately (1.0 ± 0.1) g of solid polyphenolic extracts (StymonPhen-50W) was weighed in duplicate into 50 mL Falcon tubes. Each sample was extracted with 5 mL of MeOH/H_2_O (80/20, *v*/*v*) containing 20 mg/L of butylated hydroxytoluene (BHT), vortexed and shaken for 30 min, and centrifuged at 4000× *g* rpm for 10 min. The supernatant was transferred to a 10 mL volumetric flask (protected from light). A second extraction with 5 mL of the same solvent mixture was performed, followed by shaking and centrifugation under identical conditions. Extracts were combined and brought to volume with extraction mixture. Aliquots (10 µL) of the final extract were diluted to 1:10,000; 1:25,000; and 1:50,000 in 10 mL, 25 mL, and 50 mL volumetric flasks, respectively, using 0.025% acetic acid/MeOH (90/10, *v*/*v*) as diluent. Each dilution was prepared in duplicate.

After filtration, all the samples were injected into the LC system. The monitored precursors and fragment ions, along with the different declustering potentials (DPs) and collision energies (CEs) applied, and retention times (RTs) are reported in Table 7.

**Figure 4:**
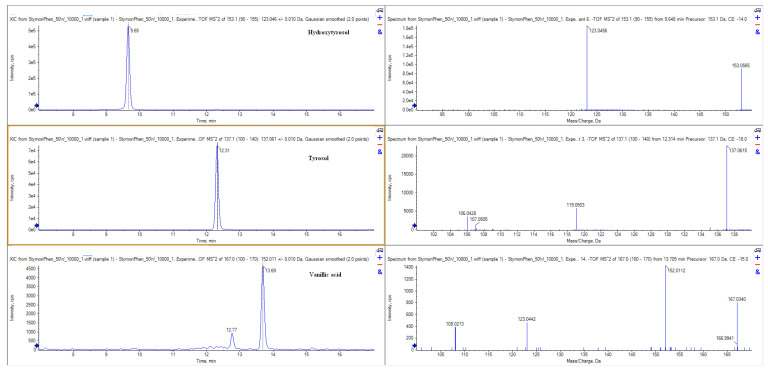
Extracted ion chromatograms (XICs) and MS/MS spectra of hydroxytyrosol, tyrosol, and vanillic acid (SCIEX 6600+ LC-QTOF) of polyphenolic extract (PPE).

The specific content of the phenolic compounds in PPE, as well as their specific chromatograms, are reported in Table 8 and Figure 4.

#### 4.1.2. Wild Boar Patty Formulation

The meat for the preparation of the patties was obtained from vacuum-packed (in packages of 4–5 kg) deboned wild boar shoulder from an approved game-handling establishment. Packs arrived within 5 days post-cutting and were used on the day of arrival. Storage was at 0–2 °C. The meat cuts were divided into appropriate portions (ca. 1.5 kg) per trial. The portions were minced (MaDo Primus, Dornhan, Germany) through a 3 mm hole plate. The ground meat was divided for carrying out two different trials, with three replicates each. The raw patties weighed approximately 60 g and had a diameter of 10 cm.

In the first trial, we produced three different formulations: control patty (CTR)-only minced meat, without antioxidant, whereas a “low PPE” (LPPE) batch and a “high PPE” PPE (HPPE) batch were prepared by adding two levels of polyphenolic extract, 1% and 2%, respectively.

In the second trial we produced four different formulations where meat patties were manufactured with a combination of PPE (0 or 2%) and sodium chloride (0 or 1.5% NaCl) as follows: CTR (without NaCl and without PPE), GA (with 1.5% NaCl and without PPE), GB (without NaCl and with 2% PPE), and GC (with 1.5% NaCl and 2% PPE).

### 4.2. Description of the Trials

Two set of trials were performed and then replicated.

Trial 1: In the first experiment, we studied proximate composition, water activity, and pH of freshly prepared wild boar meat patties with 0 (control CTR), 1% PPE (LPPE) and 2% PPE (HPPE) added. Moisture (drying at 102 °C for 3 h), crude protein (nitrogen content as determined by Kjeldahl method multiplied by 6.25), crude fat (ether extract), and ash (wet ashing method) were determined according to German official methods [38,39,40,41]. Determination of pH and water activity (a_w_) were performed on raw patties as described previously [42]. In brief, three measurements per sample were taken with a penetrating electrode for pH (Testo 205; Testo AG, Titisee-Neustadt, Germany) and with a capacitance sensor device (Lab-Swift, Novasina, Lachen, Switzerland) for a_w_ measurement. Per sample, the average was reported. Water activity data was obtained at 22.0 °C (as reported by the instrument during measurements).

Raw patties were immediately collected for initial determinations and then stored vacuum-packaged in plastic bags (Combivac 90 µm; Felzmann, Linz, Austria) for 3 and 5 days at 3 ± 1 °C and then subjected to pH determination, colour measurement, and microbiological examination, in order to establish to what extent the PPE concentration would retard growth of contaminant bacteria and extend shelf life. Samples of raw patties with 0% PPE were also collected and analyzed before vacuum packaging to define the starting characteristics of the product. Colour (CIE L*, a*, b*) [43] was measured in non-heated samples after “blooming”, i.e., keeping the meat patties wrapped in one layer of cling foil for 1 h at 3 °C to allow oxygenation of the hemoglobin. Per sample, measurements were taken at 5 spots and 3 patties were tested per treatment condition. We used a double-beam spectrophotometer with the following settings: aperture size of 8 mm, illumination of 6500 K, and observer angle of 10° (Phyma Codec 400, Phyma, Gießhübl, Austria). Hue value (arctan(b*/a*)) and saturation index or chroma [(a*)^2^ + (b*)^2^)^0.5^] were also calculated.

Delta-E [ΔE = [(L* − L*0)^2^ + (a* − a*0)^2^ + (b* − b*0)^2^]^0.5^] [44] was calculated to describe the “distance” between colours of two samples and related to day 0 of preservation within each group. A ΔE value of 1 is the smallest colour difference distinguishable to the human eye [44]. In practice, ΔE around 2 is indicative for differences perceivable only for trained assessors, and consumers would identify a difference in colours when ΔE exceeds 3.5, whereas a ΔE above 5 equals two different colours [44]. Only non-heated samples were tested.

For the microbiological examination, a 10 g aliquot of the sample was collected from 3 patties and each placed in sterile bags, and nine parts of Maximum Recovery Diluent (MRD) (Oxoid, Basingstoke, UK) were added. Homogenisation of the sample was achieved by a Stomacher-type blender (Interscience, St. Nom, France) and subsequently, tenfold serial dilutions were prepared in MRD. Samples were subjected to the following analyses: total aerobic colony count (ACC) (according to ISO 4833-2:2013 [45] on Plate Count Agar, Merck, Darmstadt, Germany, incubated for 72 h at 30 °C); *Enterobacteriaceae* count (EB) (according to ISO 21528-2:2017 [46] on Violet Red Bile Glucose Agar, Merck, incubated for 24 h at 37 °C), and *Pseudomonas* (PS) count (GSP agar, Merck, incubated for 72 h at 25 °C) [47]. The number of colony-forming units (cfus) per gram was converted to Log cfu/g.

We also studied changes in TBARSs of such stored patties after heating and subsequent storage for 1 day at 3 °C under aerobic conditions, to simulate cold storage of leftover patties to consume them the next day. Patties were heated to an internal temperature of 75 °C on a plate grill (Turbo Super Quick Grill II GG; J. Zimmermann, Oberlienz, Austria) and temperature was measured with a testo 105 thermometer (Testo AG, Titisee-Neustadt, Germany). TBARSs were determined according to Witte, Krause, and Bailey [48]. Reagents were obtained from Merck (Darmstadt, Germany). All the analyses were performed in duplicate and the average reported. Trial 1 was replicated three times.

Trial 2: In the second set of experiments, we studied colour, TBARSs, pH, a_w_, and the evolution of the microflora and textural properties (texture profile analysis, TPA) in wild boar meat patties manufactured with a combination of PPE (0 or 2%) and sodium chloride (0 or 1.5% NaCl) with the same methods previously reported. The 4 groups were therefore CTR (without NaCl and without PPE), GA (with 1.5% NaCl and without PPE), GB (without NaCl and with 2% PPE), and GC (with 1.5% NaCl and 2% PPE). Texture profile analysis (TPA) was conducted using a CT3 texture analyser, equipped with a 50 N load cell (Ametek Brookfield, Middleboro, MA, USA). From the heated patties, samples with a 30 mm diameter were punched out with a cork borer. Samples were placed between the plate of the texture analyser and a 36 mm diameter cylindrical probe. Device settings were speed 2 mm/s, 50% compression, and 2 s between the two cycles. The following parameters were calculated: hardness (N), toughness (mJ), springiness, and chewiness (N). The number of replicates was 8, and the average values ± standard deviation were reported. The water-holding capacity of the patties was assessed through cooking loss. Samples were weighed prior to cooking at 80 °C for 1 h in plastic bags. After cooling under running tap water for 30 min, they were weighed again. The cooking loss was calculated as follows: 100 × (initial weight − final weight)/initial weight.

### 4.3. Statistical Analyses

The statistical analyses were performed by ANOVA using the GLM procedure. Tukey’s post hoc test to discriminate among means were used (Statgraphics 3.0, Statgraphics Technologies, The Plains, VA, USA). Statistical significance was established at *p* < 0.05.

## Figures and Tables

**Table 1:** Proximate composition of raw wild boar meat patties with 0, 1, and 2% polyphenolic extract (PPE) added.

PPE (g/100 g)	Moisture (g/100 g)	Crude Protein (g/100 g)	Crude Fat (g/100 g)	Ash (g/100 g)
CTR	72.2 ^c^ ± 0.3	21.4 ^a^ ± 0.3	4.2 ^a^ ± 0.3	1.2 ^a^ ± 0.2
LPPE	71.6 ^b^ ± 0.3	21.3 ^a^ ± 0.2	4.3 ^a^ ± 0.2	1.1 ^a^ ± 0.1
HPPE	70.9 ^a^ ± 0.3	21.0 ^a^ ± 0.2	4.2 ^a^ ± 0.3	1.2 ^a^ ± 0.1

Samples were tested after manufacture, before storage. Each value represents the average of 3 measurements. Values expressed in mean ± standard error. Within columns, different superscripts (a, b, c) indicate statistically significant differences, *p* < 0.05.

**Table 2:** Colour parameters of raw wild boar meat patties after 0, 3, and 5 days of storage.

Colour		CTR			LPPE			HPPE		SEM		*p* Value	
	0 Days	3 Days	5 Days	0 Days	3 Days	5 Days	0 Days	3 Days	5 Days		Group	Time	GxT
L* value	40.04 ^a^	42.51 ^b^	43.29 ^c^	40.10 ^a^	42.06 ^ab^	42.90 ^bc^	40.34 ^a^	41.06 ^ab^	41.66 ^ab^	0.440	0.032	<0.001	0.179
a* value	14.25 ^c^	13.10 ^b^	12.95 ^ab^	13.37 ^b^	14.12 ^bc^	14.90 ^c^	12.37 ^a^	13.69 ^b^	14.81 ^c^	0.309	0.018	0.002	<0.001
b* value	13.37 ^b^	13.18 ^b^	13.21 ^b^	12.61 ^a^	13.48 ^b^	14.12 ^c^	12.19 ^a^	13.40 ^b^	14.21 ^c^	0.181	0.595	<0.001	<0.001
Saturation index	19.54 ^c^	18.59 ^bc^	18.54 ^bc^	18.43 ^b^	19.54 ^c^	20.54 ^d^	17.41 ^a^	19.17 ^c^	20.55 ^d^	0.305	0.039	<0.001	<0.001
Hue angle	43.29	45.31	45.86	43.30	43.77	43.47	44.60	44.38	43.82	0.677	0.062	0.333	0.154
ΔE value	-	3.39 ^ab^	4.87 ^c^	-	2.33 ^a^	4.33 ^c^	-	3.90 ^b^	4.50 ^c^	0.362	0.030	<0.001	0.150

CTR = control group without polyphenolic extract, LPPE = group with 1% polyphenolic extract, HPPE = group with 2% polyphenolic extract, L* = lightness, a* = redness, b* = yellowness, ΔE = [(L* − L*0)^2^ + (a* − a*0)^2^ + (b* − b*0)^2^]^0.5^, SEM = standard error of the mean, GxT = group × time. Samples were tested after vacuum packaging and storage at 3 °C (*n* = 81). Within rows, different superscripts (a, b, c, d) indicate statistically significant differences, *p* < 0.05.

**Table 3:** Microbial counts of raw wild boar meat patties with 0, 1, and 2% polyphenolic extract (PPE) after 3 and 5 days of storage.

Microbial Counts (Log cfu/g)	CTR		LPPE		HPPE		SEM		*p* Value	
	3 Days	5 Days	3 Days	5 Days	3 Days	5 Days		Group	Time	GxT
ACC	6.96 ^c^	7.21 ^d^	6.45 ^b^	6.91 ^c^	6.25 ^a^	6.78 ^c^	0.068	<0.001	<0.001	0.106
*Enterobacteriaceae*	3.02 ^b^	3.43 ^c^	2.92 ^b^	3.06 ^b^	2.30 ^a^	2.33 ^a^	0.182	<0.001	0.226	0.507
*Pseudomonas* spp.	3.68 ^c^	3.61 ^c^	3.37 ^bc^	3.61 ^c^	2.96 ^a^	3.18 ^b^	0.123	<0.001	0.211	0.396

CTR = control group without polyphenolic extract, LPPE = group with 1% polyphenolic extract, HPPE = group with 2% polyphenolic extract, ACC = aerobic colony count, SEM = standard error of the mean, GxT = group × time. Samples were tested after vacuum packaging and storage at 3 °C (*n* = 36). Within rows, different superscripts (a, b, c, d) indicate statistically significant differences, *p* < 0.05.

**Table 4:** TBARSs (mg MDA/kg) of raw wild boar meat patties, with 0, 1, and 2% polyphenolic extract (PPE) added after 3 and 5 days of storage and then cooked.

TBARSs (mg MDA/kg)	CTR		LPPE		HPPE		SEM		*p* Value	
	3 Days	5 Days	3 Days	5 Days	3 Days	5 Days		Group	Time	GxT
Raw	0.261 ^bA^	0.306 ^cA^	0.224 ^aA^	0.268 ^bA^	0.225 ^aA^	0.254 ^bA^	0.006	<0.001	<0.001	0.301
Heated	1.437 ^bB^	2.011 ^cB^	0.460 ^aB^	0.446 ^aB^	0.579 ^aB^	0.530 ^aB^	0.054	<0.001	<0.001	<0.001

CTR = control group without polyphenolic extract, LPPE = group with 1% polyphenolic extract, HPPE = group with 2% polyphenolic extract, TBARSs = thiobarbituric acid-reactive substances, MDA = malondialdehyde, SEM = standard error of the mean, GxT = group × time. Samples were tested after vacuum packaging and storage at 3 °C for 3 and 5 days and then heated to an internal temperature of 75 °C (*n* = 36). Within rows (a, b, c) and columns (A, B), different superscripts indicate statistically significant differences, *p* < 0.05.

**Table 5:** Microbial counts of raw wild boar meat patties with 0 and 2% polyphenolic extract (PPE) and 0 and 1.5% NaCl after 3 and 5 days of storage.

	Groups											
Microbial Counts (log cfu/g)	CTR		GA		GB		GC		SEM	*p* Value		
	3 Days	5 Days	3 Days	5 Days	3 Days	5 Days	3 Days	5 Days		Group	Time	GxT
ACC	6.80 ^e^	7.65 ^g^	6.33 ^c^	7.26 ^f^	6.01 ^b^	6.91 ^e^	5.41 ^a^	6.62 ^d^	0.077	<0.001	<0.001	0.094
*Enterobacteriaceae*	4.31 ^c^	4.81 ^d^	3.83 ^c^	4.77 ^d^	3.31 ^a^	3.68 ^b^	3.27 ^a^	3.63 ^b^	0.027	<0.001	<0.001	<0.001
*Pseudomonas* spp.	4.70 ^e^	5.71 ^g^	4.44 ^d^	5.59 ^f^	3.83 ^b^	4.26 ^c^	3.71 ^b^	3.58 ^a^	0.043	<0.001	<0.001	<0.001

CTR = group without polyphenolic extract and NaCl, GA = group without polyphenolic extract and with 1.5% NaCl, GB = group with 2% polyphenolic extract and without NaCl, GC = group with 2% polyphenolic extract and 1.5% NaCl, ACC = total aerobic colony count, SEM = standard error of the mean, GxT = group × time (*n* = 48). Within rows, different superscripts (a, b, c, d, e, f, g) indicate statistically significant differences, *p* < 0.05.

**Table 6:** Textural characteristics of wild boar meat patties, with 0 and 2% polyphenolic extract (PPE) and 0 and 1.5% NaCl, stored for 3 or 5 days at 3 °C, then heated and stored for 1 day at 3 °C.

	Groups											
Texture Profile Analyses	CTR		GA		GB		GC		SEM	*p* Value		
	3 Days	5 Days	3 Days	5 Days	3 Days	5 Days	3 Days	5 Days		Group	Time	GxT
Hardness (N)	29.32 ^a^	99.61 ^d^	38.78 ^a^	116.26 ^e^	31.89 ^a^	82.12 ^c^	51.64 ^b^	136.24 ^f^	3.252	<0.001	<0.001	<0.001
Toughness (mJ)	87.05 ^a^	350.02 ^b^	130.50 ^a^	547.22 ^c^	72.87 ^a^	329.80 ^b^	137.35 ^a^	625.12 ^c^	26.58	<0.001	<0.001	<0.001
Springiness	0.917 ^b^	0.742 ^a^	0.955 ^b^	0.867 ^ab^	0.922 ^b^	0.763 ^a^	0.935 ^b^	0.770 ^a^	0.040	0.211	<0.001	0.684
Chewiness (N)	18.33 ^a^	37.54 ^b^	27.78 ^a^	42.28 ^b^	20.79 ^ab^	30.93 ^b^	36.98 ^b^	38.17 ^b^	4.940	0.043	0.002	0.227
Cooking Loss (%)	10.53 ^a^	13.53 ^bc^	11.54 ^ab^	14.14 ^cd^	10.09 ^a^	12.69 ^b^	13.19 ^b^	15.81 ^d^	0.590	<0.001	<0.001	0.982

CTR = group without PPE and NaCl, GA = group without PPE and with 1.5% NaCl, GB = group with 2% PPE and without NaCl, GC = group with 2% PPE and 1.5% NaCl. Each value represents the average of 8 measurements (*n* = 48). Within columns, different superscripts (a, b, c, d, e, f) indicate statistically significant differences, *p* < 0.05.

**Table 7:** Retention times (RTs) and monitored ions acquired using LC-QTOF in multiple reaction monitoring.

N°	Analyte	RT (Min)	Molecular Formula	Precursor Ion (*m*/*z*)	Fragment Ion (*m*/*z*)	DP (V)	CE (V)
1	Hydroxytyrosol	9.6	C_8_H_10_O_3_	153.0557	123.0455	80	14
2	Tyrosol	12.3	C_8_H_10_O_2_	137.0608	119.0520	90	18
3	Vanillic acid	13.7	C_8_H_8_O_4_	167.0350	152.0111	70	15
4	Vanillin	14.9	C_8_H_8_O_3_	151.0401	136.0166	60	14
5	*p*-coumaric acid	15.4	C_9_H_8_O_3_	163.0401	119.0500	60	14
6	Verbascoside	16.5	C_29_H_36_O_15_	623.1981	161.0251	90	38
7	Oleuropein	17.3	C_25_H_32_O_13_	539.1770	307.0824	100	27
8	Pinoresinol	17.9	C_20_H_22_O_6_	357.1344	151.0410	80	20
9	Luteolin	18.0	C_15_H_10_O_6_	285.0405	133.0293	110	36
10	Oleuropein aglycone	18.1	C_19_H_22_O_8_	377.1242	307.0824	80	14
11	Apigenin	18.2	C_15_H_10_O_5_	269.0456	117.0343	110	35

**Table 8:** Mean contents (mg/g) of polyphenols recovered in the PPE *.

Extract	Hydroxytyrosol (mg/g)	Tyrosol (mg/g)	Vanillic Acid (mg/g)	Sum (mg/g)
PPE *	16.9	4.78	0.24	21.9

* PPE = Polyphenols extract = StymonPhen-50W.

## Data Availability

Data are made available by the authors.
